# Supplementary material for: A pyocin-like T6SS effector mediates bacterial competition in Yersinia pseudotuberculosis
Source: Microbiol Spectr. 2024 May 7;12(6):e04278-23. doi: 10.1128/spectrum.04278-23 (PMC11237486; doi:10.1128/spectrum.04278-23)
Supplement: Supplemental material — Fig. S1 to S8; Table S1 and S2. [file spectrum.04278-23-s0001.docx]

**Supplementary Information**

**A pyocin-like T6SS effector mediates bacterial**

**competition in *Yersinia pseudotuberculosis***

Leilei Yang^1^, Shuangkai Jia^1^, Sihuai Sun^1^, Lei Wang^1^, Bobo Zhao^1^, Mengsi Zhang^1^, Yanling Yin^1, 2^, Mingming Yang^1, 3^, Alex M. Fulano^4^, Xihui Shen^1, 2^, Junfeng Pan^1^* and Yao Wang^1^*

^1^State Key Laboratory for Crop Stress Resistance and High-Efficiency Production, Shaanxi Key Laboratory of Agricultural and Environmental Microbiology, College of Life Sciences, Northwest A&F University, Yangling, Shaanxi 712100, China

^2^College of Life Sciences, Tarim University, Alar 843300, Xinjiang, China

^3^State Key Laboratory for Crop Stress Resistance and High-Efficiency Production, College of Plant Protection, Northwest A&F University, Yangling, Shaanxi 712100, China

^4^Department of Plant Science and Crop Protection, University of Nairobi, P.O Box 30197-00100, Nairobi, Kenya

***For correspondence:**

Yao Wang [(wangyao@nwsuaf.edu.cn)](about:blank)

Junfeng Pan (panjf@nwsuaf.edu.cn)

**This PDF file includes:**

Supplementary Figure 1-8

Supplementary Tables 1-2


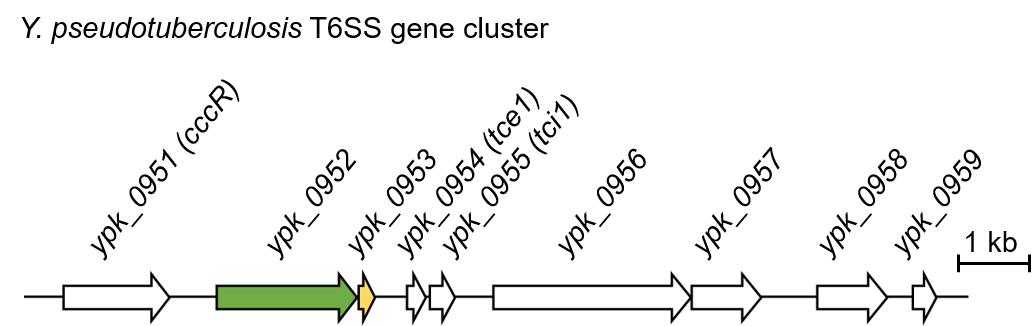


**Supplementary Figure 1. Schematic of a gene cluster encoding T6SS effectors in *Y. pseudotuberculosis*.** The arrows indicate the position and transcription direction of each gene on the T6SS gene cluster. Locus tag numbers are provided on the top of each gene, and the genes *ypk_0952* and *ypk_0953* are represented by green and yellow, respectively.


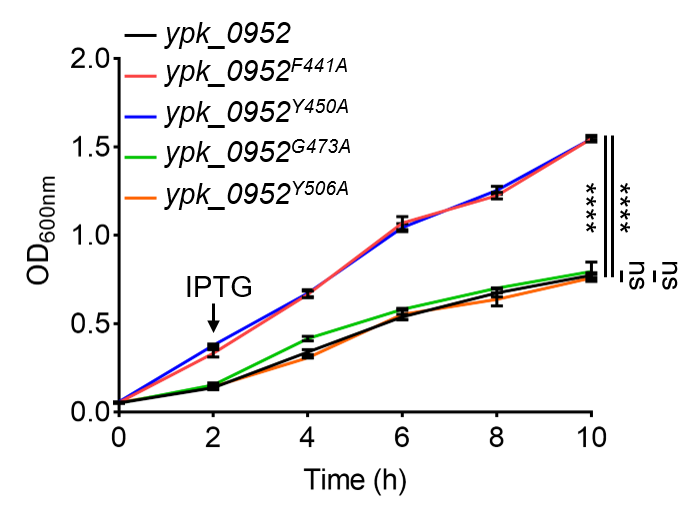


**Supplementary Figure 2.** **Growth curves of *E. coli* BL21 (DE3) harboring indicated plasmids.** The growth of the indicated strains in LB was monitored by measuring OD_600_ at indicated time points. Error bars represent the mean ± standard deviation (SD) of three independent experiments, ordinary one-way ANOVA with Tukey’s multiple comparison test with *ypk_0952*. **P* < 0.0332; ***P* < 0.0021; ****P* < 0.0002, *****P* < 0.0001; ns, not significant.


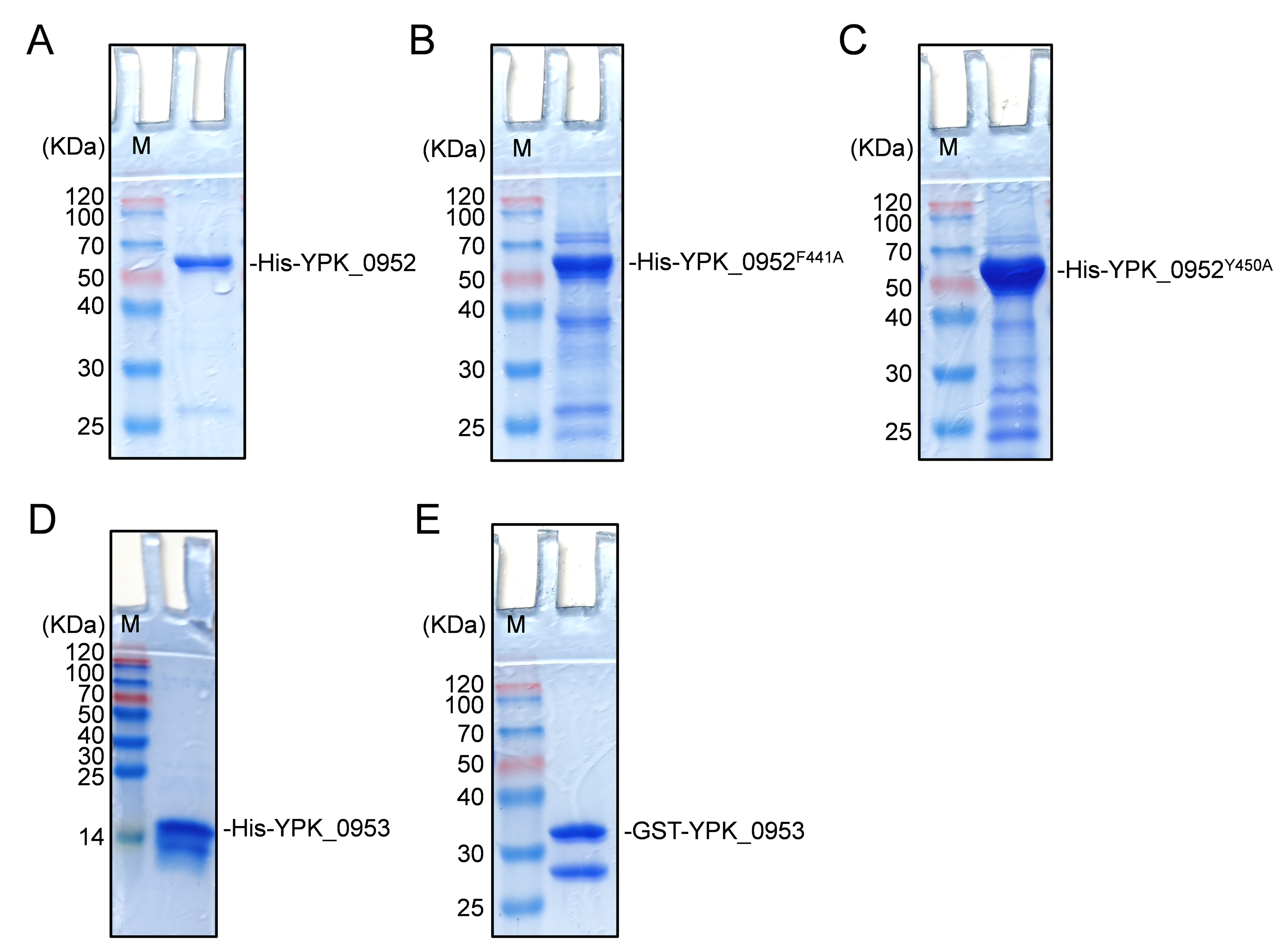


**Supplementary Figure 3:** **SDS-PAGE gels of purified His_6_-YPK_0952, His_6_-YPK_0952^F441A^, His_6_-YPK_0952^Y450A^,** **His_6_-YPK_0953, and GST-YPK_0953.** (A-D) His_6_-YPK_0952, His_6_-YPK_0952^F441A^, His_6_-YPK_0952^Y450A^, and His_6_-YPK_0953 proteins expressed in *E. coli* were purified to homogeneity using Ni^2+^-NTA affinity and analyzed with SDS-PAGE. (E) GST-YPK_0953 protein expressed in *E. coli* were purified to homogeneity using Pierce™ glutathione agarose affinity and analyzed with SDS-PAGE.

**
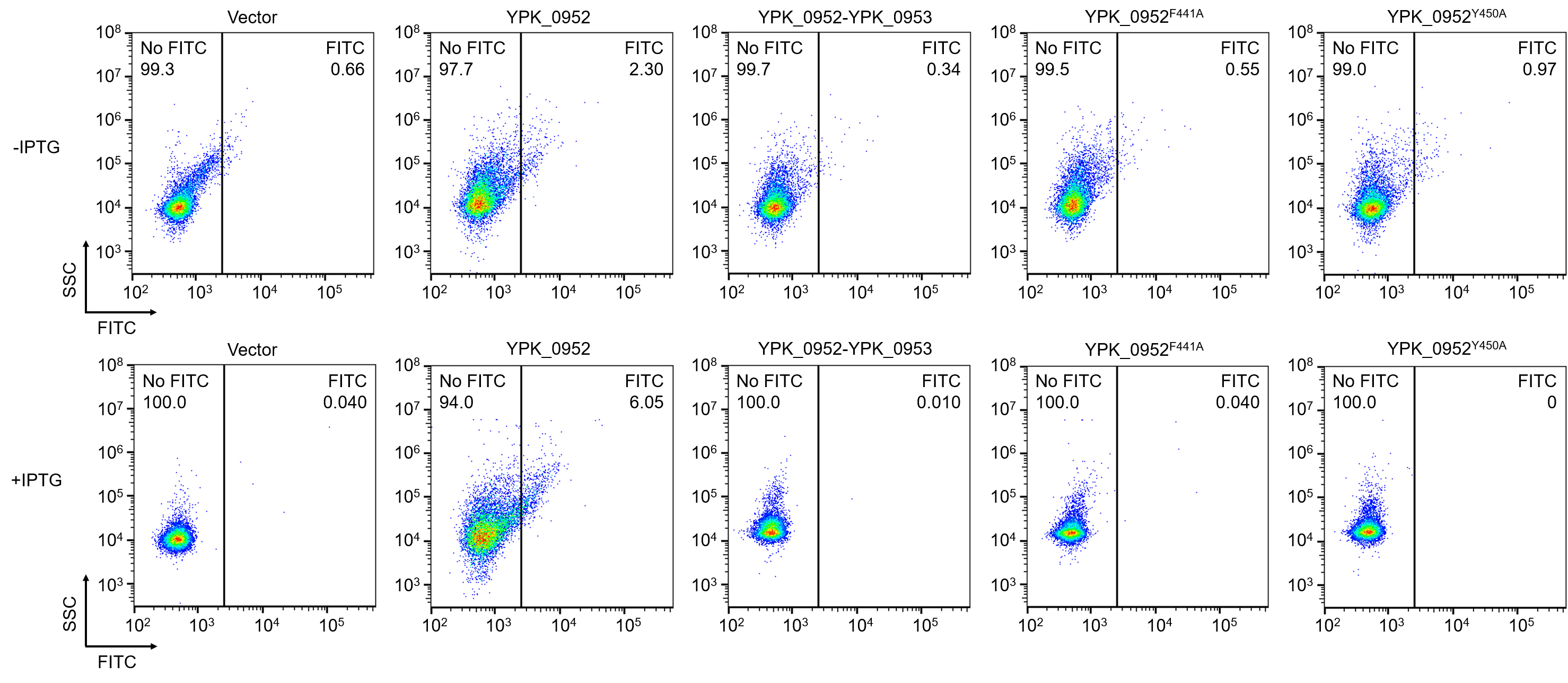
**

**Supplementary Figure 4:** **Flow cytometry experiment FITC gating diagrams.** In each figure, the bacterial morphology and size are similar, with the same gating strategy applied (-IPTG, 0 h; +IPTG, induced by IPTG for 4 h).


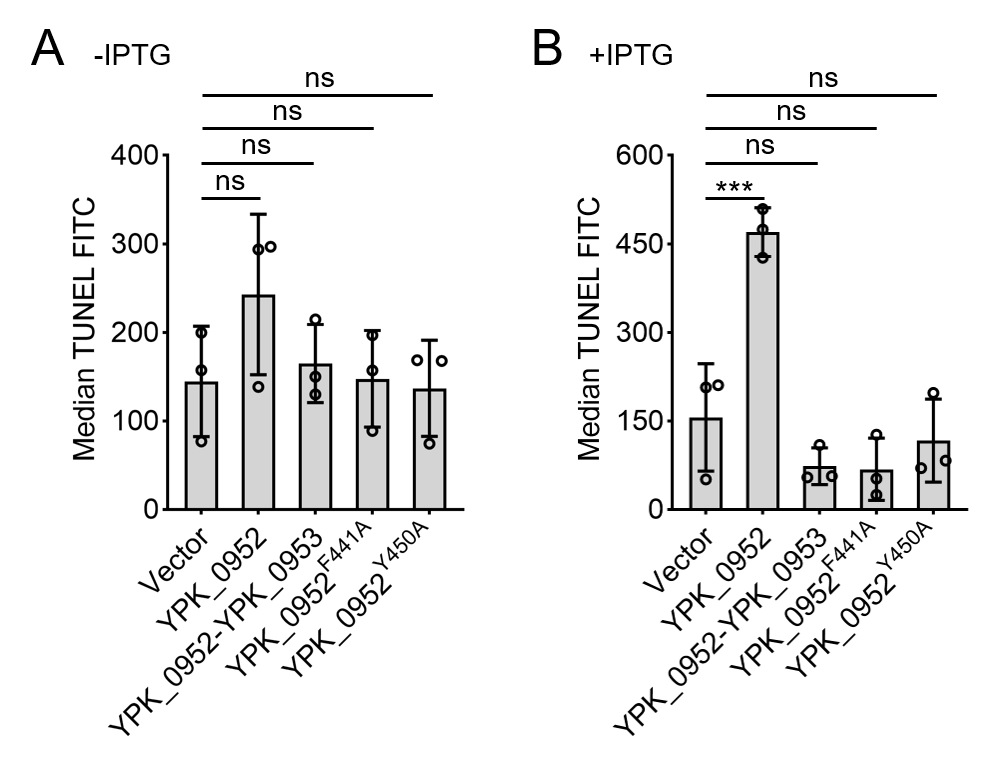


**Supplementary Figure 5:** **Flow cytometry experiment median TUNEL FITC.** (A) Flow cytometry analysis of the median TUNEL FITC of DNA fragments before IPTG induction. (B) Flow cytometry analysis of the median TUNEL FITC of DNA fragments after 4 hours of IPTG induction. Error bars represent the mean ± SD of three independent experiments, ordinary one-way ANOVA with Tukey’s multiple comparison test with Vector. **P* < 0.0332; ***P* < 0.0021; ****P* < 0.0002, *****P* < 0.0001; ns, not significant.

**
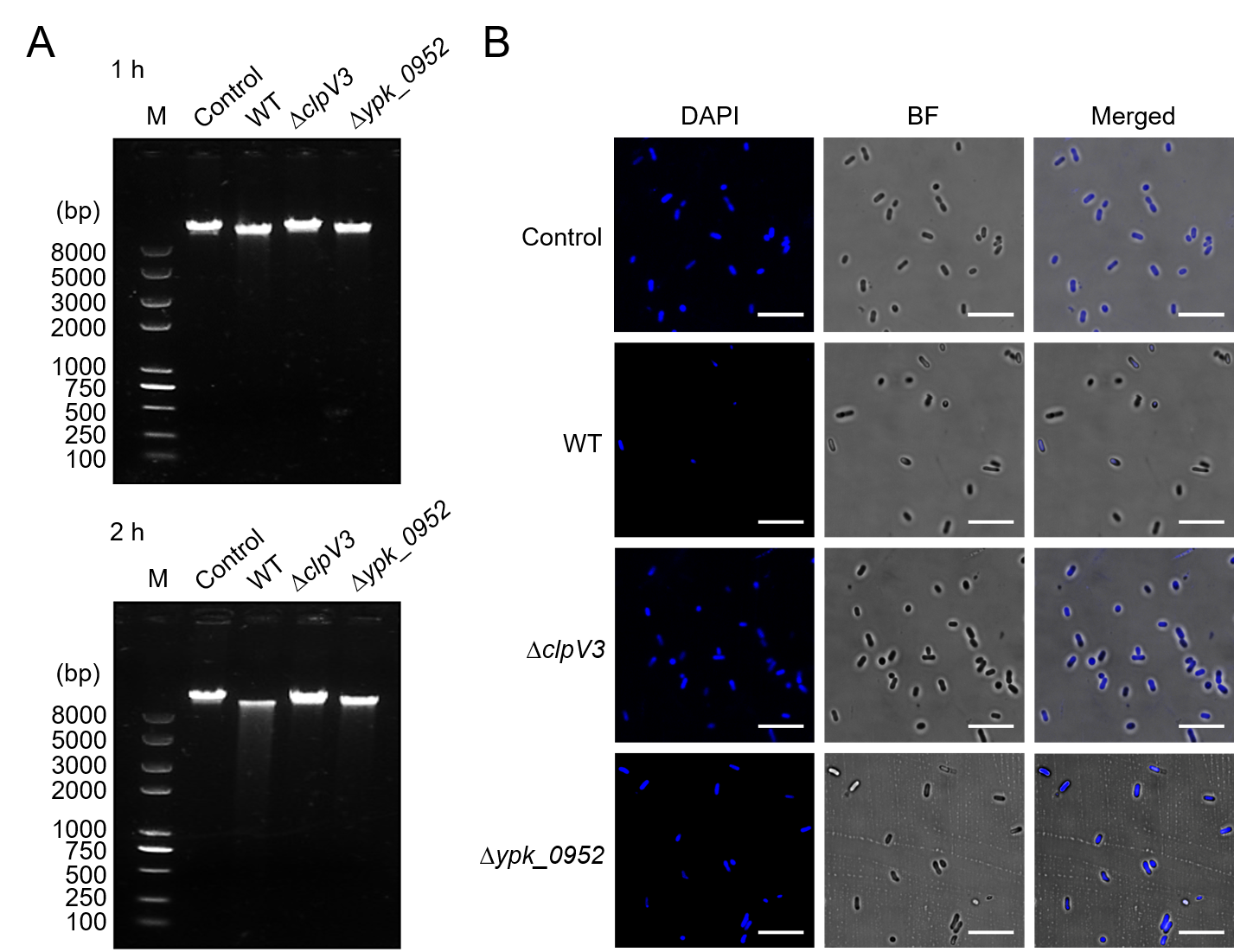
**

**Supplementary Figure 6:** **The ability of WT, ∆*clpV3*, and ∆*ypk_0952* to degrade DNA.** (A) Capacity of WT, ∆*clpV3*, and ∆*ypk_0952* sterile supernatants to degrade DNA in vitro. The sterile supernatants (13 μL) of WT, ∆*clpV3*, and ∆*ypk_0952* strains were incubated with λ DNA (0.35 μg). The products were analyzed using agarose gel. All gel results were independently repeated three times with similar outcomes. (B) YPK_0952 functions as DNase in vivo. The sterile supernatants (5 mL) of WT, ∆*clpV3*, and ∆*ypk_0952* strains were incubated with DH5α and the activity of YPK_0952 in the cells was observed using fluorescence microscopy, M9 medium as a negative control. All micrographs were independently repeated three times with similar results. DAPI, fluorescence observation; BF, bright field. Scale bar: 10 μm.


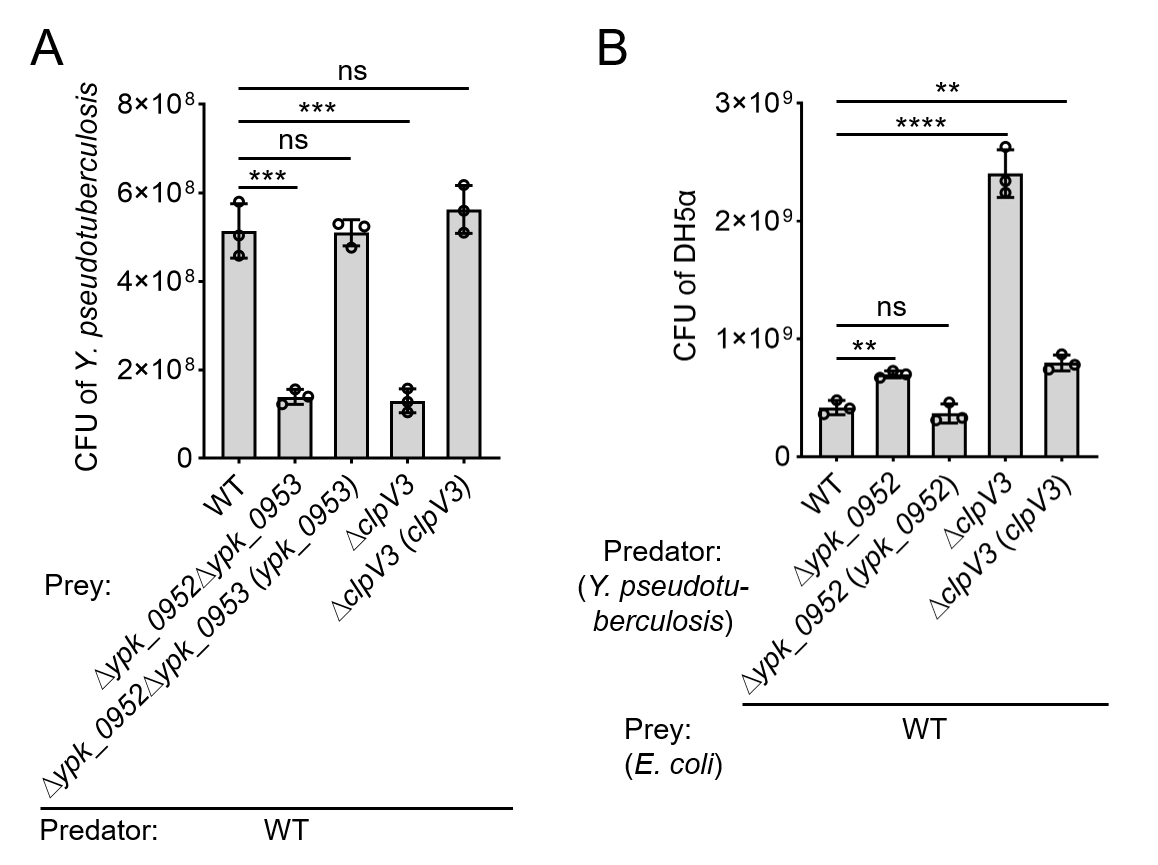


**Supplementary Figure 7:** **YPK_0952 mediates contact-independent T6SS killing.** (A) Contact-independent intra-species growth competition experiments performed by physically separating the indicated *Y. pseudotuberculosis* predator and prey strains with a membrane, and culturing them on the surface of solid medium at 26 °C for 48 hours. The CFU of the prey strains was measured based on plate counts. (B) Contact-independent Inter-species growth competition experiments performed by physically separating the indicated *Y. pseudotuberculosis* predator and *E. coli* DH5α prey strains with a membrane, and culturing them on the surface of solid medium at 26 °C for 24 hours. The CFU of the prey strains were measured based on plate counts. Error bars represent the mean ± SD of prey CFU from three independent experiments, with two-tailed, unpaired Student’s *t*-test. **P* < 0.0332; ***P* < 0.0021; ****P* < 0.0002, *****P* < 0.0001; ns, not significant.


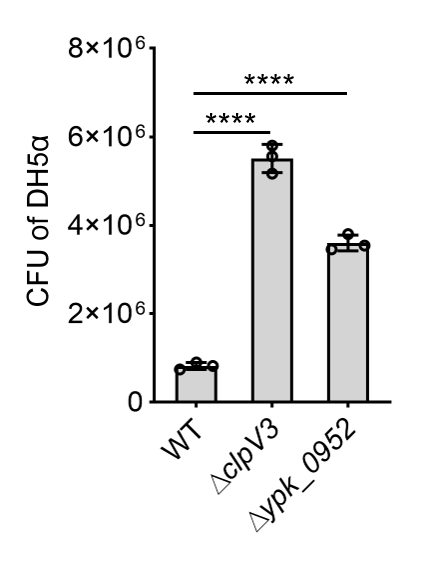


**Supplementary Figure 8: The antibacterial properties of WT, ∆*clpV3*, and ∆*ypk_0952*.** The sterile supernatants (5 mL) of WT, ∆*clpV3*, and ∆*ypk_0952* strains were co-incubated with DH5α at 37 °C for 4 hours, followed by serial dilution of the cultures and plating on LB agar plates. The CFU of DH5α strains were measured based on plate counts. Error bars represent the mean ± SD of DH5α CFU from three independent experiments, with two-tailed, unpaired Student’s *t*-test. **P* < 0.0332; ***P* < 0.0021; ****P* < 0.0002, *****P* < 0.0001; ns, not significant.

**Supplementary Table 1 Bacterial strains and plasmids used in this study**

| **Strain or plasmid** | **Relevant Characteristics** | **Reference** |
| --- | --- | --- |
| ***E. coli*** |  |  |
| S17-1 *λ pir* | *λ*-pir lysogen of S17-1, *thi pro hsdR hsdM^+^ recA* RP4 2-Tc::Mu-Km::Tn7 | [1](#_ENREF_6) |
| BL21(DE3) | Host for expression vector pET28a | Novagen |
| BTH101 | Host for bacterial two-hybrid | Novagen |
| DH5α | For competition assay | Beyotime |
| ***Y. pseudotuberculosis*** |  |  |
| WT | WT *Y. pseudotuberculosis* pIB1, Nal^R^ | 2 |
| Δ*ypk_0952* | *ypk_0952* gene deleted in *Y. pseudotuberculosis*, Nal^R^ | This study |
| Δ*ypk_0952*(*ypk_0952*) | Δ*ypk_0952* containing pKT100-*ypk_0952*, Nal^R^, Km^R^ | This study |
| Δ*ypk_0952*Δ*ypk_0953* | *ypk_0952* and *ypk_0953* gene deleted in *Y. pseudotuberculosis*, Nal^R^ | This study |
| Δ*ypk_0952*Δ*ypk_0953*(*ypk_0952*) | Δ*ypk_0952*Δ*ypk_0953* containing pKT100-*ypk_0952*, Nal^R^, Km^R^ | This study |
| Δ*ypk_0952*Δ*ypk_0953*(*ypk_0953*) | Δ*ypk_0952*Δ*ypk_0953* containing pKT100-*ypk_0953*, Nal^R^, Km^R^ | This study |
| Δ*clpV3* | *clpV3* gene deleted in *Y. pseudotuberculosis*, Nal^R^ | 3 |
| Δ*clpV3*(*clpV3*) | Δ*clpV3* containing pKT100-*clpV3*, Nal^R^, Km^R^ | 3 |
| **Plasmid** |  |  |
| pKT100 | Cloning vector, p15A replicon, Km^R^ | 4 |
| pKT100-*ypk_0952* | *ypk_0952* under the control of chloramphenicol resistance gene promoter in plasmid pKT100, Km^R^ | This study |
| pKT100-*ypk_0953* | *ypk_0953* under the control of chloramphenicol resistance gene promoter in plasmid pKT100, Km^R^ | This study |
| pACYC184 | Cloning vector, p15A origin of replication, Cm^R^ | 5 |
| pKT25 | p15A origin of replication encoding CyaA_1-224_, Km^R^ | 6 |
| pUT18C | ColE1 origin of replication encoding CyaA_225-399_, Amp^R^ | 6 |
| pKT25-*ypk_0952* | *ypk_0952* expressed in pKT25, Km^R^ | This study |
| pUT18C-*ypk_0953* | *ypk_0953* expressed in pUT18C, Amp^R^ | This study |
| pET28a | Expression vector with N-terminal hexahistidine affinity tag, Km^R^ | Novagen |
| pET28a-*ypk_0952* | pET28a carrying *ypk_0952* coding region, Km^R^ | This study |
| pET28a-*ypk_0953* | pET28a carrying *ypk_0953* coding region, Km^R^ | This study |
| pET28a-*ypk*_*0952-ypk_0953* | pET28a carrying *ypk_0952* and *ypk_0953* coding region, Km^R^ | This study |
| pGEX6p-1 | Expression vector with N-terminal GST tag, Amp^R^ | Novagen |
| pGEX6p-1-*ypk_0953* | pGEX6p-1 carrying *ypk_0953* coding region, Amp^R^ | This study |
| pDM4 | Suicide vector, *mob*RK2, *ori*R6K, *pir*, *sacB*, Cm^R^ | 7 |
| pDM4-Δ*ypk_0952* | Construct used for in-frame deletion of *ypk_0952*, Cm^R^ | This study |
| pDM4-Δ*ypk_0953* | Construct used for in-frame deletion of *ypk_0953*, Cm^R^ | This study |
| pDM4-Δ*ypk_0952*Δ*ypk_0953* | Construct used for in-frame deletion of *ypk_0952* and *ypk_0953*, Cm^R^ | This study |

*Nal^R^, Cm^R^, Km^R^, and Amp^R^ represent resistance to nalidixic acid, chloramphenicol, kanamycin, and ampicillin, respectively.

**Supplementary Table 2 Primers used in this study**

| **Primers** | **Sequence** | **Application** |
| --- | --- | --- |
| *ypk_0952-*F-*Bam*HI | CGGGATCCATGGCAAAAGGTTATTATCTGGT | To generate pET28a- *ypk_0952* |
| *ypk_0952*-R-*Eco*RI | GGAATTCTCATTGATCGTTCCAATAGTCC | To generate pET28a- *ypk_0952* |
| *ypk_0953-*F-*Bam*HI | CGGGATCCATGATTACTGAAGACTATCCATGC | To generate pET28a- *ypk_0953* and pGEX6p-1-*ypk_0953* |
| *ypk_0953*-R-*Eco*RI | GGAATTCTTATATAACCTTTCGTCCTTGC | To generate pET28a- *ypk_0953* and pGEX6p-1-*ypk_0953* |
| *ypk_0952-ypk_0953-*F-*Bam*HI | CGGGATCCATGGCAAAAGGTTATTATCTGGT | To generate pET28a- *ypk_0952-ypk_0953* |
| *ypk_0952-ypk_0953*-R-*Eco*RI | GGAATTCTTATATAACCTTTCGTCCTTGC | To generate pET28a- *ypk_0952-ypk_0953* |
| *ypk_0952-*F-*Bam*HI | GCGGGCTGCAGGGTCGACTCTAGAGGATCCATGGCAAAAGGTTATTATCTGGTTGTGG | To generate pKT25- *ypk_0952* |
| *ypk_0952-*R-*Eco*RI | TCACGACGTTGTAAAACGACGGCCGAATTCTCATTGATCGTTCCAATAGTCCATTTTATCG | To generate pKT25- *ypk_0952* |
| *ypk_0953-*F-*Bam*HI | CGCCACTGCAGGTCGACTCTAGAGGATCCCATGATTACTGAAGACTATCCATGCCC | To generate pUT18C- *ypk_0953* |
| *ypk_0953-*R-*Eco*RI | ACCATATTACTTAGTTATATCGATGAATTCTTATATAACCTTTCGTCCTTGCTTGTAGGC | To generate pUT18C- *ypk_0953* |
| *ypk_0952^F441A^*-F | GACATTGGAAAAAACATGGTTCTGAAGCTCCAGAACTAACTAATTCGAAAG | To generate pET28a- *ypk_0952^F441A^* |
| *ypk_0952^F441A^*-R | CTTTCGAATTAGTTAGTTCTGGAGCTTCAGAACCATGTTTTTTCCAATGTC | To generate pET28a- *ypk_0952^F441A^* |
| *ypk_0952^Y450A^*-F | GAACTAACTAATTCGAAAGAGGCTGTCGATGCGACTCACGATTTTG | To generate pET28a- *ypk_0952^Y450A^* |
| *ypk_0952^Y450A^*-R | CAAAATCGTGAGTCGCATCGACAGCCTCTTTCGAATTAGTTAGTTC | To generate pET28a- *ypk_0952^Y450A^* |
| *ypk_0952-*M1F*-Sph*I | CATGCATGCTTCTTAAGCGGACCATTAGC | To generate pDM4-Δ*ypk_0952* |
| *ypk_0952-*M1R | CATTATTGTGTGGGTCGGTT | To generate pDM4-Δ*ypk_0952* |
| *ypk_0952-*M2F | AACCGACCCACACAATAATGTGCCCCAAGGACTATGTTT | To generate pDM4-Δ*ypk_0952* |
| *ypk_0952-*M2R*-Sal*I | ACGCGTCGACTCGTTGAGCCAGCAATTAAA | To generate pDM4-Δ*ypk_0952* |
| *ypk_0952-ypk_0953-*M1F*-Bam*HI | CGCGGATCCGGGGGTAATCAATGAGACGG | To generate pDM4-Δ*ypk_0952*Δ*ypk_0953* |
| *ypk_0952-ypk_0953-*M1R | CCATTATTGTGTGGGTCGGT | To generate pDM4-Δ*ypk_0952*Δ*ypk_0953* |
| *ypk_0952-ypk_0953-*M2F | ACCGACCCACACAATAATGGATATAACCGGTAGGGGGATCTG | To generate pDM4-Δ*ypk_0952*Δ*ypk_0953* |
| *ypk_0952-ypk_0953-*M2R*-Sal*I | ACGCGTCGACTACGTTGGTCACGCTCTATC | To generate pDM4-Δ*ypk_0952*Δ*ypk_0953* |
| *ypk_0952-*F-*Pst*I | GAAGCTAAAATGGCATGCACTAGTCTGCAGATGGCAAAAGGTTATTATCT | To generate pKT100- *ypk_0952* |
| *ypk_0952*-R-*Bam*HI | TCTTAGTTACTTAGGTACCCGGGGATCCTCATTGATCGTTCCAATA | To generate pKT100- *ypk_0952* |
| *ypk_0953-*F-*Pst*I | AACTGCAGATGATTACTGAAGACTATCCATGC | To generate pKT100- *ypk_0953* |
| *ypk_0953*-R-*Bam*HI | CGGGATCCTTATATAACCTTTCGTCCTTGC | To generate pKT100- *ypk_0953* |

*Underlined sites indicate restriction enzyme cutting sites added for cloning.

**Reference**

1. Simon R, Priefer U, Pühler A. 1983. A Broad Host Range Mobilization System for *In Vivo* Genetic Engineering: Transposon Mutagenesis in Gram Negative Bacteria. *Bio/Technology* 1:784-791.

2. Rosqvist R, Skurnik M, Wolf-Watz H. 1988. Increased virulence of *Yersinia pseudotuberculosis* by two independent mutations. *Nature* 334:522-524.

3. Wang T, Si M, Song Y, Zhu W, Gao F, Wang Y, Zhang L, Zhang W, Wei G, Luo Z, Shen, X. 2015. Type VI Secretion System Transports Zn^2+^ to Combat Multiple Stresses and Host Immunity. *PLOS Pathog.* 11:e1005020-e1005044.

4. Hu YB, Lu P, Wang Y, Ding LS, Atkinson S, Chen SY. 2009. OmpR positively regulates urease expression to enhance acid survival of *Yersinia pseudotuberculosis*. *Microbiology* 155:2522-2531.

5. Chang AC, Cohen SN. 1978. Construction and characterization of amplifiable multicopy DNA cloning vehicles derived from the P15A cryptic miniplasmid. *J. Bacteriol.* 134:1141-1156.

6. Karimova G, Pidoux J, Ullmann A, Ladant D. 1998. A bacterial two-hybrid system based on a reconstituted signal transduction pathway. *Proc. Natl. Acad. Sci. U.S.A.* 95:5752-5756.

7. Milton DL, Otoole R, Horstedt P, WolfWatz H. 1996. Flagellin A is essential for the virulence of *Vibrio anguillarum*. *J. Bacteriol.* 178:1310-1319.
